# To Tap or Not to Tap? A Still Unanswered Question for Lumbar Puncture and Treatment Strategies in Asymptomatic Pediatric Papilledema: A Narrative Review

**DOI:** 10.3390/pediatric18050123

**Published:** 2026-09-22

**Authors:** Martina Testaì, Silvia Marino, Martina Rizza, Federico Motta, Carmela Gammeri, Giovanna Russo, Milena La Spina

**Affiliations:** 1 Unit of Pediatrics and Pediatric Emergency Care, University Hospital Policlinico “G. Rodolico-San Marco”, University of Catania, 95100 Catania, Italy; martinat617@gmail.com (M.T.); silvia_marino86@hotmail.it (S.M.); federicomotta14@gmail.com (F.M.); melaniagammeri96@gmail.com (C.G.); milena.laspina@unict.it (M.L.S.); 2 School of Medicine, University of Catania, 95100 Catania, Italy; martina.rizza02@gmail.com; 3 Pediatric Hematology Oncology Unit, University Hospital Policlinico “G. Rodolico-San Marco”, University of Catania, 95100 Catania, Italy

**Keywords:** papilledema, idiopathic intracranial hypertension, lumbar puncture

## Abstract

Papilledema is a potentially vision-threatening finding in children, often diagnosed during routine ophthalmological evaluation in asymptomatic subjects, which requires prompt diagnostic evaluation to exclude intracranial pathology. The available literature suggests that a significant proportion of cases with confirmed idiopathic intracranial hypertension (IIH) may present without headache or other manifestations of increased intracranial pressure. Current diagnostic criteria for pediatric IIH include lumbar puncture (LP) as a mandatory step following neuroimaging. However, the invasive nature of this procedure raises critical questions regarding the identification of patients who truly require this investigation. Indeed, the challenge of distinguishing true papilledema from pseudopapilledema represents the critical diagnostic prerequisite, requiring an expert pediatric neuro-ophthalmologist to ensure appropriate patient selection and avoid unnecessary tests. Evidence regarding the management of asymptomatic IIH is limited. Most published cohorts report treatment with acetazolamide despite the absence of symptoms, probably based on concerns regarding potential visual deterioration rather than prospective data. Currently, data from pediatric populations characterizing the natural history of untreated patients are lacking, and there is no consensus regarding treatment duration, tapering strategies, or long-term follow-up for this group. This narrative review summarizes the current literature on the diagnostic and therapeutic strategies for asymptomatic pediatric papilledema. It focuses on the role of lumbar puncture, the importance of differentiating papilledema from pseudopapilledema, and the uncertainties surrounding treatment decisions. Nevertheless, significant knowledge gaps persist regarding the optimal management of these patients, highlighting the need for prospective studies.

## 1. Introduction

Papilledema is defined as optic disc swelling secondary to elevated intracranial pressure (ICP) [1]. It may be associated with a broad spectrum of disorders, including intracranial tumors, hydrocephalus, cerebral venous sinus thrombosis, craniosynostosis, central nervous system infections, inflammatory syndromes, and idiopathic intracranial hypertension (IIH) [2,3,4], a condition first coined as pseudotumor cerebri syndrome (PTCS) by Nonne in 1904 [5]. Consequently, recognition of papilledema generally prompts an urgent diagnostic work-up aimed at identifying potentially life-threatening causes of intracranial hypertension.

Among the various etiologies, IIH represents one of the most common causes [2], although it remains a rare disorder, with an estimated annual incidence of approximately 0.6–0.9 cases per 100,000 children [6,7,8]. Epidemiologically, the condition differs by pubertal status: post-pubertal patients share a risk factor profile similar to that of adults, notably female sex and obesity [9,10,11,12], whereas pre-pubertal individuals exhibit a more balanced distribution, with a female-to-male ratio of nearly 1:1 [13,14,15].

Clinical manifestations are highly heterogeneous. Headache remains the most frequently reported symptom [7,16,17]; although, diplopia, transient visual obscuration, tinnitus, nausea or vomiting, and sixth cranial nerve palsy may also occur [14,18,19,20]. Visual involvement requires careful evaluation, as permanent visual impairment represents the most serious long-term complication of the disease [13,14,15,21,22,23,24,25]. Notably, a significant proportion of children may present without headache or visual complaint despite the presence of elevated intracranial pressure, with the diagnosis being established following routine ophthalmological examination [13,18,19,26,27,28,29,30], raising important questions regarding the optimal diagnostic and therapeutic approach in this specific subgroup. Although a formal consensus definition is currently lacking in pediatric guidelines, in clinical practice asymptomatic papilledema generally refers to the incidental detection of optic nerve head edema secondary to intracranial hypertension in a patient exhibiting no systemic or visual symptoms.

Diagnostic criteria employed in most contemporary pediatric studies are based on revised Friedman criteria, published in 2013 [31], which refined and expanded the historical modified Dandy criteria [32] for the diagnosis of PTCS. According to current recommendations, patients require neuroimaging to exclude secondary causes of intracranial hypertension, followed by lumbar puncture (LP) with cerebrospinal fluid (CSF) analysis and measurement of CSF opening pressure. As such, LP remains a cornerstone of the diagnostic pathway in children with suspected IIH [33]. Nevertheless, the invasive nature of the procedure continues to generate debate regarding its role, particularly in asymptomatic patients and in cases where the diagnosis is uncertain. For this reason, a critical step in the diagnostic pathway is the accurate differentiation between true papilledema and pseudopapilledema, which may mimic optic nerve head swelling in children. Misclassification may lead either to unnecessary invasive investigations or, conversely, to a missed diagnosis of intracranial hypertension and, this distinction, consequently, often requires expert pediatric neuro-ophthalmological assessment [34,35,36].

Beyond diagnostic uncertainty, therapeutic decision-making remains equally challenging. Although acetazolamide represents the first-line pharmacological treatment following a diagnosis of idiopathic intracranial hypertension [18,33,37], its use in patients without symptoms is largely driven by concerns regarding potential visual deterioration and the hypothesis that this presentation may represent an early stage of disease [26,28,38], rather than robust prospective evidence. Currently, there is no consensus in clinical practice regarding treatment strategies, their duration or tapering protocols, or long-term follow-up for this cohort [18].

The purpose of this review is to critically analyze the available evidence regarding diagnostic pathway and therapeutic decision-making in asymptomatic pediatric papilledema, with particular attention to the distinction between papilledema and pseudopapilledema, current diagnostic criteria, the role of lumbar puncture, therapeutic strategies and existing gaps in knowledge.

## 2. Materials and Methods

A narrative review of the literature was conducted using PubMed/MEDLINE as the primary database, supplemented by Embase and Scopus. The search was performed until June 2026, without publication date restrictions, in order to include both historical papers and contemporary evidence. The search strategy was conducted using combinations of the terms “papilledema,” “idiopathic intracranial hypertension,” “pseudotumor cerebri syndrome,” “children,” “pediatric,” “lumbar puncture,” “opening pressure,” “asymptomatic,” and “pseudopapilledema,” using Boolean operators (AND/OR) and adapting the search syntax to each database. Additional relevant articles were identified through manual reference screening of selected publications.

Studies were considered eligible if they included pediatric patients (≤18 years) and focused on papilledema, suspected papilledema, pseudopapilledema, PTCS or IIH. Particular attention was given to studies describing asymptomatic presentations and discussing diagnostic and therapeutic strategies in pediatric patients. Eligible publications included original articles, retrospective cohort studies, case series, reviews and consensus papers.

Publications were excluded if they included only adult patients, did not evaluate pediatric papilledema, pseudopapilledema, PTCS or IIH or did not provide information relevant to the diagnostic or therapeutic questions addressed in this review.

The literature search was performed independently by two authors and combined for article writing. Any uncertainties regarding study eligibility were discussed with the other authors and resolved by consensus. This review was not designed as a systematic review or meta-analysis; therefore, no formal quality assessment or quantitative synthesis was performed.

## 3. Papilledema or Pseudopapilledema? The First Diagnostic Challenge

### 3.1. Etiologies of Pediatric Papilledema

Identification of papilledema in children requires timely evaluation, as it may reflect a wide spectrum of potentially serious neurological disorders. Recent studies have shown that the relative frequency of the underlying etiologies varies substantially according to clinical setting, highlighting the importance of contextualizing the diagnostic process [2,39] (Table 1).

Less common conditions (incidence < 5%) include: hematological and oncological disorders, such as acute myeloid leukemia (4%) and B-cell acute lymphoblastic leukemia (1%); immune-mediated diseases (e.g., ADEM, lupus cerebritis, MOGAD, and Behçet’s disease); infections like Lyme disease and systemic or structural anomalies such as malignant hypertension, intracranial abscesses, and Chiari I malformation [2,39].

### 3.2. Pseudopapilledema and Why Misdiagnosis Matters

Pseudopapilledema represents a group of congenital or structural optic disc abnormalities that mimic the appearance of papilledema in the absence of raised intracranial pressure. In the pediatric population, the most common causes include optic disc drusen (ODD), variably calcified extracellular hyaline deposits in the optic disc head found in approximately 0.4% to 1% of this age group [41,42], as well as tilted optic nerves (commonly seen in myopia), small crowded optic nerves (especially in hyperopic eyes) and other developmental anomalies of the optic nerve head [43,44]. 

Because these conditions may present with blurred optic disc margins and an elevated disc appearance, distinguishing pseudopapilledema from true papilledema can be challenging, particularly in mild cases where examination findings may overlap [43]. It is especially relevant for ODD, which are more difficult to detect in children than in adults because they are more frequently buried, thus obscured by overlying tissue [45], and non-calcified, limiting the utility of imaging techniques [44]. Specifically, buried ODD, masked by overlying optic nerve head tissue, may lack the typical ophthalmoscopic appearance of superficial drusen, and appear as an elevated optic disc with blurred margins that can closely mimic papilledema [44,45]; non-calcified ODD may further limit the sensitivity of techniques such as B-scan ultrasonography, which relies partly on the acoustic signal produced by calcified deposits [44]. In this context, enhanced-depth imaging optical coherence tomography (EDI-OCT) could be the most effective method to evaluate ODD in pediatric patients; however, this test is rather difficult because of the limited collaboration of children [46,47].

This distinction is of critical importance because overdiagnosis may expose children to unnecessary investigations, including neuroimaging, hospitalization, lumbar puncture, and medical treatment, generating avoidable risks, anxiety, and healthcare costs [34,48]. Conversely, misdiagnosing true papilledema as pseudopapilledema may delay the diagnosis of potentially serious intracranial disorders, increasing the risk of neurological complications and permanent visual impairment [43,44].

Differentiating these two entities represents one of the most significant clinical issues in pediatric neuro-ophthalmology and remains a crucial step in the diagnostic pathway of patients presenting with optic disc elevation.

Optic neuritis should also be considered in the differential diagnosis of optic disc swelling, as children are more likely to exhibit papillitis [49], an inflammatory involvement of the optic nerve head. This condition is typically associated with objective visual dysfunction, such as reduced visual acuity, dyschromatopsia, an afferent pupillary defect, or a central visual field defect and may be accompanied by pain, particularly with eye movements [49].

Therefore, before proceeding with invasive procedures, careful assessment is required to determine whether the disc edema represents true papilledema.

### 3.3. The Role of Neuro-Ophthalmological Assessment

Given the potential consequences of both over- and under-diagnosis, accurate evaluation of optic disc swelling requires expert neuro-ophthalmological consultation to guide subsequent management decisions.

Funduscopic examination remains the cornerstone of this process. Clinical features suggestive of true papilledema include optic disc hyperemia, obscuration of the disc margins, hemorrhages, cotton wool spots and Paton lines [50]. Conversely, findings such as visible optic disc drusen, anomalous vascular branching patterns and presence of spontaneous venous pulsations may indicate pseudopapilledema [33]. Nevertheless, substantial overlap exists, particularly in cases of mild papilledema or buried optic disc drusen, making clinical examination alone insufficient and often requiring additional investigations [33,43,44,51].

For this reason, ancillary imaging techniques are increasingly incorporated into diagnostic work-up. Optical coherence tomography (OCT) is a non-invasive technique that provides quantitative assessment of optic nerve head morphology and retinal nerve fiber layer (RNFL) thickness. It can be useful both for the diagnosis and longitudinal follow-up of papilledema but its diagnostic performance in children varies according to the parameters assessed, and RNFL thickness alone may not reliably distinguish papilledema from pseudopapilledema [44]. More specific OCT features, including Bruch’s membrane configuration or presence of retinal or choroidal folds, may provide additional discriminatory information [44]. In a pediatric cohort, a combination of RNFL thickness and width of Bruch’s membrane classified eyes with papilledema versus pseudopapilledema with a sensitivity of 91.7%, specificity of 92.2% [52].

EDI-OCT may provide additional information by detection of buried optic disc drusen: in a comparative study it shows the highest diagnostic accuracy (sensitivity 95%, accuracy 97%) [53].

Conversely, B-scan ultrasonography may be particularly useful when OCT acquisition is limited by poor cooperation, as it requires less patient fixation. However, its diagnostic performance may be limited in children because optic disc drusen are frequently non-calcified [54]. In a pediatric cohort, it achieved 74% accuracy in differentiating these two conditions [55].

Fundus autofluorescence may also assist in the detection of ODD, particularly when the drusen are superficial, whereas its sensitivity may be lower for buried lesions [44]. Therefore, newly developed OCT techniques appear promising for supporting differential diagnosis and follow-up of these patients [56].

Nevertheless, a recent American Academy of Ophthalmology technology assessment concluded that, due to the lack of high-quality evidence supporting the utility of any single ophthalmic imaging method to differentiate pediatric papilledema from pseudopapilledema, no gold standard diagnostic test currently exists [51]. A comprehensive clinical approach remains the definitive strategy, combining detailed history-taking and expert ophthalmological examination with the judicious use of auxiliary tests to determine which children require further evaluation.

## 4. Lumbar Puncture: Is It Always Necessary?

Having reasonably established the presence of true papilledema, the focus shifts to the next critical question: is lumbar puncture always necessary to confirm or exclude PTCS?

### 4.1. Current Diagnostic Criteria

The diagnosis of pediatric pseudotumor cerebri syndrome relies on a combination of clinical signs and symptoms, neuroimaging findings, and cerebrospinal fluid analysis. Historically, diagnostic criteria were derived directly from adult cohorts and then adapted to pediatric patients, as seen with the modified Dandy Criteria proposed by Smith in 1985 [32], which were long considered the standard references and were used in a multicenter clinical trial [57]. However, a paradigm shift occurred in 2013 with the publication of criteria proposed by Friedman et al. [31], which introduced specific pediatric parameters that are now more universally accepted among pediatric neuro-ophthalmologists [33]. Moreover, recognizing that pediatric population exhibits unique features and distinct epidemiological profiles across different age groups, Aylward respected this stratification by proposing specific standards tailored for pre-pubertal children, in order to avoid the underdiagnosis of younger patients, who generally present a lower accepted normal opening pressure [58].

To clarify the evolution of these concepts, Table 2 compares the three cited classifications: the historically adapted modified Dandy criteria [32], the widely adopted Friedman criteria [31] and the age-stratified pre-pubertal Criteria proposed by Aylward [58].

Each of these diagnostic criteria has distinct clinical relevance and specific advantages depending on the pediatric subgroup. The modified Dandy criteria, derived from adult cohorts, lack specific cutoff values for normal opening pressure in younger children, carrying a risk of misdiagnosis in pre-pubertal patients. The 2013 Friedman criteria substantially mitigated these limitations by establishing definitive pediatric CSF opening pressure thresholds and integrating neuroimaging parameters, the widely accepted standard for research and clinical practice. Meanwhile, Aylward’s age-stratified recommendations offer a distinct advantage in pre-pubertal cohorts. Overall, rather than strictly applying a single rigid scheme to all age groups, current evidence supports an individualized diagnostic approach.

Interestingly, in some patients, the diagnosis of IIH was based by the presence of papilledema and typical headache, together with clear clinical improvement following CSF drainage, despite an opening pressure below the diagnostic threshold [59]. Moreover, Tibussek et al. highlighted that CSF opening pressure measurements in children may be influenced by several procedural factors including pain, stress, patient movement, body position, needle position, and sedation or analgesia [60]. These findings suggesting that a single manometric measurement reflects only a static moment in a dynamic physiological system and demonstrating that the overall clinical context should always guide diagnosis rather than a strict numerical cut-off.

### 4.2. Evidence from Asymptomatic Pediatric Cohorts

Although headache and other symptoms of raised intracranial pressure are considered the most common manifestations of pediatric IIH, a non-negligible proportion of children may be completely asymptomatic at diagnosis, with papilledema identified incidentally during routine ophthalmological examinations.

Retrospective analyses have highlighted significant variability in prevalence: Bassan et al. reported that 31% of their cohort was asymptomatic [28], while Gondi et al. identified a proportion of 22% [26]. Furthermore, Whitecross et al. described 19% of cases as presenting without symptoms at diagnosis [30], and Aylward et al. reported that 5% of their total sample (representing 36% of those without headache) were asymptomatic [27].

Despite the absence of subjective symptoms, diagnostic management in these cohorts has always included a lumbar puncture to confirm clinical suspicion [26,27,28,29,30], consistently applied diagnostic pathways that include invasive investigations as a mandatory component. In this context, LP serves two essential purposes: documenting intracranial hypertension through opening pressure measurement by manometry, and analyzing CSF composition, which is essential for ruling out inflammatory, infectious, or neoplastic conditions. A significantly lower opening pressure was observed by Gondi et al. in asymptomatic children compared to their symptomatic counterparts [26], whereas Bassan et al. found no significant difference, reporting similar mean values between the two groups [28] (Table 3).

Currently, in accordance with the accepted diagnostic criteria [31,32,58] scientific literature does not support a definitive diagnosis of IIH without inclusion of LP. Consistent with these criteria, our review of the available studies showed that all asymptomatic cohorts incorporated LP into their diagnostic pathway [26,27,28,29,30]. Although the diagnostic approach was uniform across studies, they did not provide concordant evidence regarding opening pressure values between symptomatic and non-symptomatic patients.

Therefore, the standard diagnostic work-up follows a well-defined sequential path: it begins with the ophthalmological evaluation that raises the clinical suspicion of papilledema, proceeds with neuroradiological investigation (MRI or CT) to exclude space-occupying lesions or other intracranial pathologies, and concludes with the execution of a lumbar puncture for the measurement of opening pressure and CSF analysis [18,19,33] (Figure 1).

## 5. Treatment Strategies in Asymptomatic Pediatric Papilledema

Once the diagnosis of IIH has been confirmed, the management of asymptomatic children with papilledema remains one of the most controversial aspects of clinical practice. Unlike symptomatic patients, in whom treatment is generally aimed at controlling symptoms, reducing optic disc swelling and preventing visual deterioration [33], the optimal approach to children presenting with papilledema in the absence of headache, or other manifestations of raised intracranial pressure, remains uncertain.

### 5.1. General Therapeutic Principles

Treatment for PTCS consists of medical therapy as primary approach, whereas surgical intervention is reserved for refractory cases or for patients with rapidly progressive visual impairment. Although there are no randomized controlled trials specifically for the pediatric population, the Idiopathic Intracranial Hypertension Treatment Trial (IIHTT) evaluated the use of acetazolamide combined with weight reduction versus diet and placebo, in adults aged 18–60 years with mild visual loss. Findings from this study supported the use of acetazolamide to achieve improvements in papilledema and visual acuity [57]. In pediatric practice, it is the first-line therapy, and the standard dosage is 15–25 mg/kg/day, administered in two or three divided doses. Depending on the clinical course and symptom severity, this may be increased to 100 mg/kg/day, with a maximum of 2 g/day in children and 4 g/day in adolescents [33].

Topiramate is also used in children, as second line option for those who cannot tolerate acetazolamide. Recommended dose, for children aged 6 to 12 years, is 15 mg once daily, with an increase to a target dose of 2 to 3 mg/kg/day divided twice daily (maximum 200 mg/day) [33]. Furosemide tends to be used if first-line treatment is not tolerated or as adjunctive therapy, at a dose of 0.3–0.6 mg/kg/day [19]. Likewise, corticosteroids are not routinely recommended and are generally reserved for selected situations characterized by rapidly progressive visual deterioration or as a temporary bridge to definitive interventions [61]. Surgical intervention is generally reserved for children with rapidly progressive visual impairment or severe or worsening papilledema despite maximal medical therapy [13,33]. Reported procedures include CSF diversion through ventriculoperitoneal or lumboperitoneal shunting, optic nerve sheath fenestration [33] and cerebral venous sinus stenting [19]. CSF shunting is generally considered when a rapid reduction in intracranial pressure is required, particularly in those with severe visual loss [62]. However, the available evidence is predominantly observational [62], and shunt-related complications, including obstruction, infection, CSF leak, and the need for revision, are important considerations in the pediatric population, which may be at increased risk of such complications.

Optic nerve sheath fenestration may be considered when visual loss is the predominant concern, or when patients are considered unsuitable for more invasive procedures [19]. Bersani et al. reported improvement or stabilization of visual function, highlighting its efficacy and supporting its use in pediatric population [63]. The procedure carries potential risks including retinal artery occlusions, retrobulbar hemorrhage, traumatic optic neuropathy and strabismus [62].

Venous sinus stenting may represent an alternative option for carefully selected patients with refractory IIH, with the aim of improving cerebral venous outflow, reducing venous pressures and thereby facilitating resorption of CSF through arachnoid granulations. However, long-term safety and efficacy data remain limited, and the irreversible nature of stent placement must be carefully considered [19,62].

Overall, management is influenced by the level of visual loss and the severity of the clinical presentation [19] and the choice of surgical procedure should be individualized; it has been suggested that a more aggressive therapeutic approach may be warranted for younger children, as reliable follow-up is frequently difficult to obtain, as well as for those presenting with severe papilledema, which is associated with poorer visual outcomes [21,64].

Furthermore, there is no consensus regarding the optimal duration of treatment, tapering strategies, or criteria for treatment discontinuation. Similarly, follow-up protocols vary considerably among centers and have not been standardized in the pediatric population [18,22,37,38,65].

### 5.2. Evidence from Asymptomatic Pediatric Cohorts

Despite this uncertainty, most published pediatric cohorts report treatment of asymptomatic patients once the diagnosis of IIH has been established. Acetazolamide remains the first-line pharmacological therapy and is generally continued until resolution of papilledema.

Bassan et al. identified 14 asymptomatic cases, all of whom received medical treatment with acetazolamide. Complete resolution of optic disc swelling was observed with a significantly shorter median treatment duration in asymptomatic patients compared to symptomatic ones (3 vs. 6 months). One asymptomatic patient experienced a relapse [28].

Gondi et al. described a cohort of 12 asymptomatic children; 8 patients (66.7%) were managed with medical therapy. Those treated with acetazolamide required a significantly lower dosage compared to symptomatic patients (361.11 vs. 986.88 mg daily). Conversely, 4 patients did not receive treatment; among these, 2 experienced a subsequent relapse, while 1 did not. Treatment resulted in complete remission for all treated subjects [26].

Aylward et al. evaluated a cohort of 22 children presenting with optic nerve edema without headache. Within this population, 8 patients (36.4%) were incidentally identified during routine eye examinations and completely lacked clinical symptoms. Regarding management acetazolamide was used in 86.4% of non-headache group, while 22.7% tried either furosemide or topiramate and 9% required surgical treatment. Seven patients in the entire study sample (including 1 in the non-headache group) did not receive any treatment at the discretion of the neurologist, though their specific asymptomatic status was not clarified [27].

Whitecross et al. reported heterogeneous management in a series where asymptomatic patients represented 19% of the sample (13/68); 1 was treated with serial lumbar punctures and 2 required definitive surgical intervention. Chronic optic nerve edema was noted in 54% of the cohort and permanent vision loss occurred in one child [30].

Weig et al. described a case series of 3 patients who completely lacked clinical symptoms. Two were diagnosed with true IIH, while one was treated unsuccessfully with a brief course of prednisone. Notably, one case experienced a relapse after treatment discontinuation, requiring a second course of acetazolamide despite medication-related side effect, with improvement of ocular abnormalities. Therapy was ultimately discontinued and his ocular examination remained abnormal, although visual acuity remained normal [29].

From the analysis of the asymptomatic cohorts, it becomes evident how management strategies are highly heterogeneous and probably guided by the need to balance the risk of silent visual loss against the burden of overtreatment. The decision to defer therapy, as observed in the studies by Gondi et al. [26] and Aylward et al. [27], likely reflects a watchful waiting approach, aimed at avoiding medication-induced side effects. However, this conservative strategy may expose patients to inherent risks, highlighted by the recurrences reported among untreated individuals in Gondi’s cohort [26]. Nevertheless, such relapses might also reflect the variable natural history of the disease, given that Bassan et al. and Weig et al. reported recurrence even in medically treated non-symptomatic patients [28].

Conversely, recourse to surgical intervention or serial lumbar punctures, documented by Whitecross et al. [30], demonstrates that an asymptomatic presentation does not preclude severe ophthalmic progression, such as permanent visual impairment, necessitating aggressive management when visual risk is judged to be significant.

This heterogeneity extends to the choice and dosing of pharmacological agents. Acetazolamide, as the established first-line therapy, was the most widely used medication across the reviewed cohorts [26,27,28]. However, a minority of patients received alternative drugs, such as topiramate or furosemide [27,29], while prednisone showed no response [29]. In clinical practice, these agents are typically employed as second-line options for those who do not tolerate acetazolamide, whereas steroids are reserved as a temporary bridge therapy. Findings from Bassan et al. [28] and Gondi et al. [26], show that children presenting without symptoms may require significantly lower dosages and shorter treatment durations to achieve complete resolution, and suggest that clinicians should adjust the dosage to the minimal effective dose, adapting to what may be a milder disease phenotype. Ultimately, in the absence of specific pediatric guidelines, these diverse approaches reflect the clinicians’ effort to individually tailor management.

Treatment duration and criteria for therapy discontinuation were not consistently reported across publications. In the cohorts evaluated by Bassan et al. [28] and Weig et al. [29], therapy was discontinued after complete resolution of papilledema. Gondi et al. [26] and Aylward et al. [27] assessed optic disc swelling severity during ophthalmological evaluation using the Frisen grading system, whereas the specific evaluative methods were not clearly specified in the remaining reports.

Treatment durations corresponded to an approximate mean of 7.4 months for non-symptomatic patients and 11.9 months for symptomatic patients. However, these values should be interpreted cautiously, as they are derived from only two retrospective series [26,28] and therefore cannot be considered representative estimates of treatment duration in pediatric IIH.

The available evidence is summarized in Table 3, which provides limited but informative comparative data on papilledema severity, opening pressure, treatment strategies, treatment duration, and clinical outcomes.

### 5.3. Current Uncertainties

Some authors have hypothesized that asymptomatic papilledema may represent either a milder phenotype or an earlier stage of the disease [26,28]. This hypothesis is indirectly supported by observations that asymptomatic patients often require lower acetazolamide dosages and shorter treatment durations than symptomatic children [26,28]. However, a major limitation of the existing literature is the lack of prospective studies evaluating the natural history of this cohort, and available evidence remains insufficient to confirm this assumption, as the long-term outcomes of untreated cases are largely unknown.

Management remains highly heterogeneous and largely guided by clinical judgment and perceived risk of visual loss rather than by robust evidence-based recommendations. Consequently, no consensus has been reached regarding either systematic observation alone or universal pharmacological treatment of asymptomatic patients. In the absence of prospective data, this approach reflects a precautious strategy with most clinicians opting to treat in order to minimize the risk of potentially irreversible visual impairment.

## 6. Discussion

The present review highlights several critical challenges in the management of asymptomatic pediatric papilledema.

A major finding emerging from the literature is that an asymptomatic presentation is not uncommon in pediatric IIH. Across several retrospective cohorts, approximately 20- 30% of patients had neither headache nor other symptoms of raised ICP at diagnosis, with papilledema often detected incidentally during routine ophthalmological examinations [26,28,30]. In contrast, Aylward et al. found that only 5% of the overall cohort was completely asymptomatic, although these patients represented 36% of those presenting without headache [27].

This finding is clinically relevant because it demonstrates that the absence of symptoms cannot reliably exclude intracranial hypertension and reinforces the importance of careful ophthalmological assessment, particularly in children presenting with optic disc elevation. Supporting this concept, Fischer et al. in a heterogeneous cohort including both primary and secondary causes of papilledema, reported that nearly half of all cases were attributable to secondary causes of intracranial hypertension. Importantly, papilledema represented the first manifestation of previously unrecognized pathology in four asymptomatic patients (6% of the overall cohort and 11% of the IIH subgroup), all of whom were ultimately diagnosed with IIH [39].

The next critical milestone in the diagnostic pathway is distinguishing true papilledema from pseudopapilledema, a significant neuro-ophthalmological hurdle. This distinction carries profound practical implications although, according to a recent American Academy of Ophthalmology technology assessment, no gold standard diagnostic test currently exists [51].

The magnitude of this diagnostic challenge is illustrated by Kovarik et al., who reported that 76% of children referred for suspected papilledema were ultimately diagnosed with pseudopapilledema or normal optic nerves. Among the ten completely asymptomatic patients in their cohort, nine had pseudopapilledema or normal nerve variants, while one had bilateral optic nerve head drusen [48]. Similarly, Liu et al. reported that among 52 children initially referred with a suspicion of papilledema, the clinical conclusion was revised to pseudopapilledema in exactly half of the cases (26 patients) following a pediatric ophthalmological review at a tertiary care center [34]. In this context, the characteristics of headache, if present, may provide additional information when interpreting an apparently elevated optic disc. Pain associated with raised intracranial pressure is classically described as persistent or daily, worse on awakening or in the recumbent position, and exacerbated by Valsalva maneuvers [66]. Nevertheless, clinical presentations in children with PTCS can be either episodic or constant/daily, and common locations include focal pain or involvement of head/neck/shoulders [17]. Thus, the presence of headache alone should not be considered diagnostic of raised intracranial pressure, but its temporal pattern and associated features may contribute to the overall clinical assessment.

Failure to recognize true papilledema may delay the diagnosis of potentially life-threatening intracranial disorders and increase the risk of irreversible visual impairment [43,44]. Conversely, misdiagnosing pseudopapilledema as true papilledema may expose children to unnecessary neuroimaging, lumbar puncture, pharmacological treatment, hospitalization, psychological distress, and increased healthcare costs [34,35]. For example, Liu et al. documented that 53.8% of children with pseudopapilledema (14 out of 26 patients) underwent a lumbar puncture before their diagnosis was corrected, showing a statistically significant difference in CSF opening pressure compared to children with true IIH. Moreover, of patients diagnosed with pseudopapilledema, 46.1% (12 out of 26 children) were taking medication due to a misdiagnosis of IIH, including acetazolamide. Treatments were discontinued only after the diagnosis was revised [34]. Similarly, Kovarik et al. described four children who, after being referred from primary care with suspected papilledema, underwent a lumbar puncture in the hospital before a specialist examination ultimately refuted the diagnosis, revealing pseudopapilledema or normal optic nerve variants [48]. This trend is also evident in a series by Kaplan et al., where as many as 52% of children diagnosed with pseudopapilledema (30 out of 58 patients) underwent an LP [56].

To reduce unnecessary invasive investigations ancillary imaging modalities—including OCT, enhanced-depth imaging OCT, fundus autofluorescence, and ocular ultrasonography—are increasingly incorporated into the diagnostic work-up [33,43,44,56]. Although these tools offer valuable diagnostic support, no single modality has yet proven robust enough to serve as a standalone gold standard [51]. More recently, artificial intelligence has emerged as a promising frontier: Chang et al. demonstrated that a deep learning AI model evaluating pediatric fundus photographs could achieve a sensitivity of over 90% for detecting true papilledema, suggesting a potential role as an effective triage tool [67]. Nevertheless, comprehensive neuro-ophthalmological evaluation remains the indispensable cornerstone of diagnosis and clinical decision-making.

Another important issue emerging from the literature concerns the role of LP in children presenting with papilledema, particularly those who are completely asymptomatic.

The available evidence suggests that LP remains essential to fulfill current diagnostic criteria, as all published cohorts of asymptomatic pediatric IIH included CSF opening pressure measurement as part of the diagnostic work-up [26,27,28,29,30]. In this context, LP provides a dual diagnostic benefit by objectively confirming intracranial hypertension while allowing exclusion of inflammatory, infectious, and neoplastic disorders through CSF analysis.

However, an important distinction should be made between the diagnostic criteria required to establish pediatric IIH and the broader clinical question of whether every child presenting with suspected papilledema should automatically undergo an invasive procedure. While the existing literature demonstrates that CSF opening pressure measurement has traditionally been incorporated into the diagnostic pathway, this does not necessarily imply that LP should be performed indiscriminately in every child with optic disc swelling. To support this, Fischer et al. reported that only 79% of children with papilledema underwent LP; in the remaining cases, the procedure was deferred due to relative or absolute contraindication, a recent prior LP performed by an outside provider or there were clinical and radiographic signs of elevated intracranial pressure from shunt failure or a subdural hematoma [39]. Similarly, Liu et al. found that 92.3% of children diagnosed with IIH underwent CSF opening pressure measurement, indicating that even in highly specialized neuro-ophthalmological cohorts the procedure is occasionally omitted when clinically justified [34].

Consequently, LP should be deferred in the presence of recognized contraindications, including signs of raised intracranial pressure associated with an increased risk of herniation, hemodynamic shock, ongoing or recently uncontrolled seizures, significant coagulation abnormalities or thrombocytopenia (platelet count < 40 × 10^9^/L), anticoagulant therapy, local infection at the puncture site, or respiratory insufficiency [68].

These findings emphasize the importance of careful patient selection. The primary objective should be to identify patients in whom true papilledema has been confidently established, by an experienced neuro-ophthalmologist, and for whom the diagnostic benefits of LP outweigh the risks associated with an invasive procedure. Therefore, following neuroimaging and exclusion of secondary causes, according to current evidence and accepted diagnostic criteria we consider lumbar puncture as an essential component for suspected pediatric IIH, in order to confirm intracranial hypertension and complete the diagnostic evaluation, provided that no contraindications are present. This approach, conditioned by the absence of pediatric-specific guidelines and the need for a patient-tailored assessment, is further supported by the available literature in asymptomatic children, as all published cohorts incorporated this procedure into the diagnostic work-up [26,27,28,29,30].

Prospective studies addressing the natural history of untreated asymptomatic pediatric IIH would be needed to determine whether some patients may have a self-limited course and safely managed through serial clinical and ophthalmological evaluation, without invasive confirmation.

Interestingly, emerging non-invasive techniques for ICP assessment, including optic nerve sheath diameter ultrasonography, transcranial Doppler, MRI-based methods, ophthalmic approaches and otic methods, may represent promising tools for future clinical application. Among these, MRI-based ICP estimation and two-depth Doppler have shown particular potential; however, no non-invasive approaches have yet demonstrated sufficient accuracy and reliability to replace invasive ICP measurement in routine clinical practice [69]. This review also highlights considerable uncertainty regarding treatment once asymptomatic IIH has been confirmed. Most published cohorts adopted a precautionary approach, with asymptomatic patients receiving acetazolamide despite the absence of symptoms. This strategy appears to be driven primarily by concern regarding potential visual deterioration rather than by evidence-based recommendations specific to this population. Consequently, management is generally extrapolated from symptomatic IIH, where treatment aims to control symptoms, reduce papilledema, and preserve visual function [33]. Notably, as suggested by Beres et al., adult consensus guidelines may reasonably be extrapolated to children for the overall management of IIH [33,70].

The available pediatric literature indicates that medical treatment, predominantly with acetazolamide, is frequently associated with resolution of papilledema [26,28,29], although relapses have also been described [28]. Nevertheless, management remains highly heterogeneous, ranging from observation alone—which was associated with a risk of relapse in two of four untreated patients [26]—to second-line therapies such as furosemide or topiramate [27,29], serial LPs [30], and even surgical interventions [27,30].

Studies have also suggested that individuals presenting without symptoms may require lower medication doses and shorter treatment durations than symptomatic ones, raising the possibility that they represent a milder phenotype or an earlier stage of disease [26,28]. This hypothesis is further supported by the clinical data from Gondi et al., who found that this subgroup presented with a significantly lower opening pressure on LP and a significantly lower papilledema grade in both eyes [26]. However, the favorable outcome observed in treated non-symptomatic patients should be interpreted with caution, as they may be influenced by selection bias rather than attributable to treatment efficacy. Moreover, these observations remain speculative since no prospective studies have adequately characterized the natural history of untreated asymptomatic pediatric IIH. Indeed, as pointed out by Bassan et al., current data do not permit studying the true natural course of the condition or determining whether it would eventually resolve without intervention [28]. Studies reveal that most asymptomatic cases receive medical treatment [26,27,28,29], although small cohorts of untreated children have been documented [26,27]. However, adopting an observational strategy requires extensive expertise from the clinical center and highlights the need for prospective studies to clarify the safety of a watch-and-wait approach. Nonetheless, when treatment is deemed necessary, clinicians should focus on adjusting doses to the minimum required for disease control, in order to favor an individualized approach on a case-by-case basis.

The absence of standardized management extends beyond the decision to initiate treatment. Considerable variability also exists regarding treatment duration, tapering schedules, criteria for treatment discontinuation, and follow-up protocols [18,22,37,38,65]. This heterogeneity likely reflects the limited availability of high-quality pediatric evidence and the consequent reliance on retrospective studies, small case series, expert opinion, and extrapolation from adult data. As a result, therapeutic decisions continue to depend largely on clinician judgment and the perceived risk of visual deterioration.

Finally, the interpretation of the available evidence is limited by the predominantly retrospective design of published studies and their origin from tertiary referral centers, which may introduce potential selection bias. Furthermore, long-term visual outcomes are frequently insufficiently reported, and very few cohorts include untreated patients. Consequently, it remains unclear whether the favorable outcomes observed in many studies primarily reflect treatment efficacy or the intrinsically benign course of a subset of an asymptomatic case.

In conclusion, the available evidence supports a stepwise approach and therapeutic approach. Accurate differentiation between papilledema and pseudopapilledema should represent the initial diagnostic priority. Once true papilledema has been confirmed and secondary causes excluded through neuroimaging, lumbar puncture remains necessary according to current diagnostic standards for pediatric IIH. Following diagnosis, management should be individualized, carefully balancing the potential risk of visual deterioration against the limited evidence supporting treatment in asymptomatic patients.

## 7. Limitations

The findings of this review should be interpreted considering several limitations. First, this was a narrative review and no formal assessment of methodological quality or risk of bias of the included studies was performed. Second, the available pediatric literature is largely based on retrospective observational studies and small cohorts, particularly among asymptomatic patients, limiting the precision and generalizability of the findings. The retrospective nature of most studies also introduces the possibility of selection bias. Furthermore, substantial heterogeneity was observed in reporting key clinical variables, including papilledema severity, opening pressure, treatment regimens and duration, criteria for treatment discontinuation, and follow-up. This heterogeneity limits direct comparison across studies and precludes quantitative synthesis. Crucially, given the limited number of untreated asymptomatic cases, characterizing the natural history of this entity remains challenging, making it difficult to differentiate therapeutic benefits from spontaneous resolution.

These limitations substantially affect the strength of the conclusions that can be derived. In particular, the available evidence does not allow definitive recommendations regarding when LP can safely be deferred in asymptomatic children, neither does it establish the superiority of any specific therapeutic strategy. Therefore, the findings of this review should be interpreted cautiously and within the context of individualized clinical decision-making.

## 8. Clinical Practice and Future Directions

Current evidence remains insufficient to identify, with clear-cut certainty, a subgroup of asymptomatic children in whom LP can be safely omitted. Nevertheless, in daily clinical practice, a pragmatic risk–benefit approach may guide decision-making in asymptomatic children. True papilledema should first be confirmed by an experienced neuro-ophthalmologist, followed by neuroimaging to exclude secondary causes and assess for indirect signs of elevated intracranial pressure. In the presence of concerning neuroimaging findings or persistent suspicion of raised intracranial pressure, LP should be performed, provided there are no contraindications. Conversely, when the diagnosis is uncertain or clinical, ophthalmological, and neuroimaging findings are reassuring, the potential benefits of LP should be weighed against its procedural burden and risks, particularly as anesthesia may be required in children. Moreover, CSF opening pressure represents a single time-point measurement that may be influenced by physiological and procedural factors.

In selected asymptomatic children, LP may therefore be postponed following multidisciplinary assessment and provided that close clinical and ophthalmological follow-up can be ensured. This requires the child to be able to reliably report visual changes or new symptoms and the family to understand the rationale and potential risks of watchful waiting and adhere to scheduled monitoring. A strong therapeutic alliance is essential to ensure continuity of follow-up and prompt re-evaluation, including reconsideration of LP, should any clinical or ophthalmological change occur. This represents a cautious, individualized approach rather than a validated prospective algorithm.

Future research should prioritize prospective, multicenter pediatric studies specifically designed to investigate asymptomatic IIH. Key objectives include defining the natural history of untreated disease, identifying clinical, neuro-ophthalmological, and radiological predictors of progression, and establishing evidence-based criteria for treatment initiation.

Additional studies are needed to determine the optimal duration of medical therapy, tapering strategies, and follow-up protocols.

Further validation of emerging neuro-ophthalmological imaging techniques may improve diagnostic accuracy and facilitate earlier differentiation between papilledema and pseudopapilledema. Given the central role of lumbar puncture in current diagnostic criteria, future efforts should also focus on improving risk stratification to identify specific subgroups of children who are most likely to benefit from invasive diagnostic evaluation while avoiding unnecessary procedures.

Ultimately, the development of pediatric-specific diagnostic and therapeutic guidelines will require higher-quality evidence than is currently available. Until such data emerge, clinical decision-making will continue to rely largely on expert judgment and individualized risk assessment.

## 9. Conclusions

Asymptomatic papilledema represents a challenging clinical scenario in pediatric practice. Available evidence demonstrates that a substantial proportion of children with idiopathic intracranial hypertension may present without headache or other manifestations of raised intracranial pressure, with optic disc swelling identified incidentally during ophthalmological evaluation.

The first and most important step in the diagnostic pathway is the accurate differentiation between true papilledema and pseudopapilledema. This distinction is essential to avoid both delayed recognition of potentially serious intracranial disorders and unnecessary investigations in children with benign optic disc anomalies. In this setting, expert neuro-ophthalmological assessment plays a crucial role in guiding patient selection.

Current evidence supports lumbar puncture as an essential component of the diagnostic evaluation once true papilledema has been confirmed and IIH is suspected. Despite concerns regarding its invasiveness, the available literature does not currently support a definitive diagnosis of pediatric PTCS without cerebrospinal fluid opening pressure measurement and CSF analysis. Whether LP can be safely deferred in a selected subgroup of asymptomatic children remain uncertain and cannot currently be established on the basis of the available evidence. In this context, any decision to defer LP should remain highly individualized and based on a reliable multidisciplinary assessment and close longitudinal follow-up, with a low threshold for prompt re-evaluation should any clinical or ophthalmological change occur.

Management of asymptomatic pediatric IIH remains characterized by considerable uncertainty. Most published cohorts describe treatment with acetazolamide despite the absence of symptoms, largely because of concerns regarding potential visual deterioration. However, the natural history of untreated asymptomatic disease remains poorly defined, and there is currently no consensus regarding treatment initiation, duration, tapering strategies, or follow-up protocols. Consequently, management should be individualized and based on multidisciplinary assessment, balancing the potential risk of visual loss against the limited evidence supporting routine treatment in asymptomatic patients.

Future pediatric studies are needed to clarify the natural history of this condition, optimize patient selection for invasive investigations, and establish evidence-based diagnostic and therapeutic recommendations for this unique population.

## Figures and Tables

**Figure 1:**
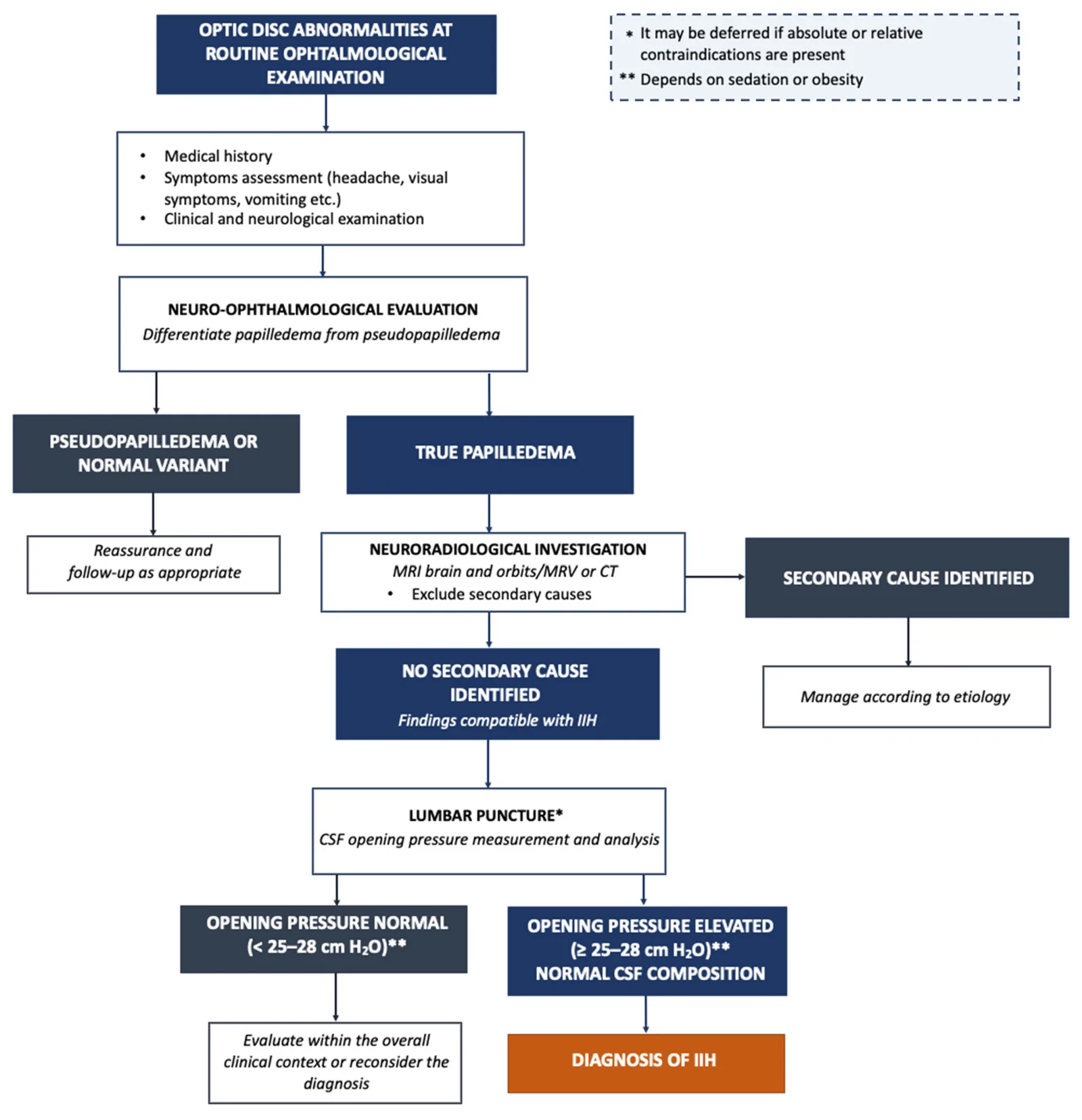
Diagnostic algorithm for pediatric papilledema. Proposed on the basis of current evidence.

**Table 1:** Etiology of pediatric papilledema according to clinical setting.

Etiology	Outpatient/Community Setting	Inpatient/Tertiary Care Setting
IIH	42.1% [2]	51% [39]
Subdural hematoma	-	83% [39]
Craniosynostosis	18.4% [2]	-
Intracranial tumors	15.8% [2]	13% [40]
Infectious disorders	-	6% [39]
Cerebral venous sinus thrombosis	-	6% [39]
Hydrocephalus/shunt malfunction	-	5.3%/1% [39]

IIH: Idiopathic intracranial hypertension.

**Table 2:** Comparison of diagnostic criteria for pediatric PTCS.

	Modified Dandy Criteria [32]	Friedman Criteria [31]	Pre-pubertal Aylward Criteria [58]
Clinical signs and symptoms	Signs/symptoms of increased intracranial pressure (headache, nausea, vomiting, transient visual obscurations, or papilledema)	A—Papilledema	5—Bilateral optic disc edema 6—Symptoms suggestive of elevated intracranial pressure (headache, nausea, vomiting, transient visual obscurations, tinnitus) that improve following CSF drainage
Neurological examination	Absence of localizing neurological signs (except for unilateral or bilateral abducens nerve palsy)	B—Normal examination, except for cranial nerve abnormalities	2—No focal neurological signs (with the exception of cranial nerve IV or VI palsy)
Neuroimaging	Normal or small ventricles visualized on CT or ventriculography	C—Normal brain parenchyma without evidence of hydrocephalus, mass, or structural lesion and no abnormal meningeal enhancement on MRI, with and without gadolinium, for typical patients (female and obese), and MRI, with and without gadolinium, and MRV for others; if MRI is unavailable or contraindicated, contrast-enhanced CT may be used	4—Exclusion of recognized secondary causes
CSF composition	Normal	D—Normal	3— Normal
CSF opening pressure	>25 cm H_2_O	E—≥250 mm CSF (≥25 cm H_2_O) if the child is non-sedated and non-obese >280 mm CSF (>28 cm H_2_O) in children	1—>18 cm H_2_O in children <8 years >25 cm H_2_O in children ≥8 years or <8 years without optic edema
Diagnostic formulation	Definitive diagnosis only	Definite: patient fulfills criteria A–E Probable: criteria A–D are met but the measured CSF pressure is lower than specified for a definite diagnosis *	Definite: patient meets criteria 1–6 Probable: Patient meets criteria 1–4 and either 5 or 6

CSF: cerebrospinal fluid; CT: computed tomography; MRI: magnetic resonance imaging; MRV: magnetic resonance venography. * Diagnosis of pseudotumor cerebri syndrome without papilledema: in the absence of papilledema, a diagnosis of pseudotumor cerebri syndrome can be made if B–E from above are satisfied, and in addition the patient has a unilateral or bilateral abducens nerve palsy. In the absence of papilledema or sixth nerve palsy, a diagnosis of pseudotumor cerebri syndrome can be suggested but not made if B–E from above are satisfied, and in addition at least 3 of the following neuroimaging criteria are satisfied: empty sella, flattening of the posterior aspect of the globe, distention of the perioptic subarachnoid space with or without a tortuous optic nerve, transverse venous sinus stenosis [31].

**Table 3:** Comparative summary of diagnostic and therapeutic findings in published cohorts of asymptomatic pediatric IIH.

Study	Asymptomatic Cohort (*n*)	Papilledema Grade	Opening Pressure	Treatments and Dose	Treatments Duration	Outcomes
Bassan et al. [28]	14	NR	Asymptomatic: median 325 mm H_2_O Symptomatic: median 320 mm H_2_O	Acetazolamide dose NR	Asymptomatic: median 3 mo Symptomatic: median 6 mo	Complete resolution of papilledema in all; treatment stopped after resolution Follow-up median 19.5 mo Relapse: 1
Gondi et al. [26]	12	Modified Frisen Grade OD mean 2.75 ± 0.4 OS mean 2.85 ± 0.51	Asymptomatic: median 342.5 ± 51 mm H_2_O Symptomatic: median 462.4 ± 131.9 mm H_2_O	Acetazolamide Asymptomatic: 361.11 ± 250 mg/day Symptomatic: 968.88 ± 564.84 mg/day Untreated: 4/12	Asymptomatic: median 11.78 mo ± 9.92 Symptomatic: median 17.77 ± 8 mo	Complete resolution of papilledema in all treated Relapse: 2
Aylward et al. [27]	8/22 of non-headache group	Frisen Grade Asymptomatic grade NR	Asymptomatic/symptomatic NR	Acetazolamide: 86.4% Topiramate/Furosemide: 22.7% Surgery: 9% Untreated: 1/22 data of non-headache group Not clear in asymptomatic	NR	NR
Whitecross et al. [30]	13	NR	Asymptomatic: median 340 mm H_2_O Symptomatic: median 370 mm H_2_O	Serial LP: 1/13 Surgery: 2/13	NR	Chronic optic nerve edema: 7/13 Permanent vision loss: 1/13
Weig et al. [29]	2	NR	Patient 2: 260 mm H_2_O Patient 3: 230 mm H_2_O	Acetazolamide in patient 2 (2 cycles) dose NR Furosemide in patient 3 dose NR Prednisone in patient 3 dose NR	NR	Patient 2: complete resolution of papilledema, treatment stopped after resolution (2 times) Relapse: 1 Not progression of papilledema in patient 3

Data are reported as provided in the original studies. NR: not reported; mo: months; OD: right eye; OS: left eye; LP: lumbar puncture.

## Data Availability

No new data were created or analyzed in this study. Data sharing is not applicable to this article.

## Abbreviations

IIH	Idiopathic intracranial hypertension
ICP	Intracranial pressure
LP	Lumbar puncture
PTCS	Pseudotumor cerebri syndrome
CSF	Cerebrospinal fluid
ADEM	Acute Disseminated Encephalomyelitis
MOGAD	Myelin oligodendrocyte glycoprotein antibody disease
ODD	Optic disk drusen
EDI-OCT	Enhanced-depth imaging optical coherence tomography
RNFL	Retinal nerve fiber layer
OCT	Optical coherence tomography
MRI	Magnetic resonance imaging
CT	Computed tomography
IIHTT	Idiopathic intracranial hypertension treatment trial
AI	Artificial intelligence
